# Machine Learning Seams of Conical Intersection: A Characteristic Polynomial Approach

**DOI:** 10.1021/acs.jpclett.3c01649

**Published:** 2023-08-24

**Authors:** Tzu Yu Wang, Simon P. Neville, Michael S. Schuurman

**Affiliations:** † Department of Chemistry and Biomolecular Sciences, University of Ottawa, Ottawa, Ontario K1N 6N5, Canada; ‡ National Research Council Canada, 100 Sussex Dr., Ottawa, Ontario K1A 0R6, Canada

## Abstract

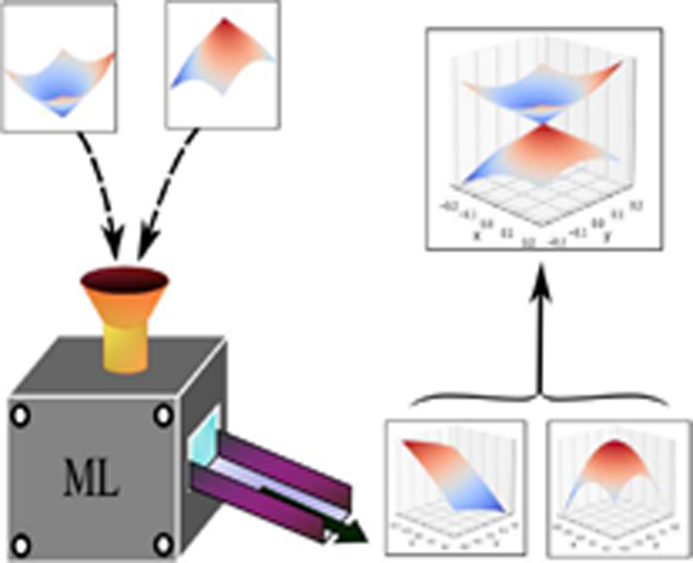

The machine learning of potential energy surfaces (PESs) has undergone rapid progress in recent years. The vast majority of this work, however, has been focused on the learning of ground state PESs. To reliably extend machine learning protocols to excited state PESs, the occurrence of seams of conical intersections between adiabatic electronic states must be correctly accounted for. This introduces a serious problem, for at such points, the adiabatic potentials are not differentiable to any order, complicating the application of standard machine learning methods. We show that this issue may be overcome by instead learning the coordinate-dependent coefficients of the characteristic polynomial of a simple decomposition of the potential matrix. We demonstrate that, through this approach, quantitatively accurate machine learning models of seams of conical intersection may be constructed.

Potential energy surfaces (PESs) are of central importance in understanding chemical processes, forming a crucial ingredient for the simulation and rationalization of the spectroscopic and dynamic properties of molecular systems. Accordingly, there exists great interest in the construction of accurate PES models fitted to *ab initio* energies. Here, recent advances in machine learning (ML) promise to be transformative,^1−5^ with accurate, high-dimensional ML models of PESs now being routinely constructed. However, there exists an important caveat to this success: the majority of applications to date have been concerned with the learning of isolated ground state PESs. If one is interested in excited state PESs, then an additional complication must be accounted for. Namely, that, in general, excited state PESs exhibit extended seams of conical intersection (CIs).^6,7^ The existence of a CI seam between two or more electronic states means that the corresponding adiabatic PESs belong to the *C*^0^ differentiability class over any domain containing it. This lack of differentiability, or nonsmoothness, of the PESs on the locus of seam points is problematic for many standard ML approaches, including kernel methods, and neural networks employing gradient-based optimization. Such models will struggle to correctly describe CI seams; in terms of topography and, more importantly, the dimensionality of the branching space. Conical intersections are not isolated points but rather form connected seams of degeneracy. At a point of two-state conical intersection, for example, this degeneracy is lifted at first-order in precisely two directions, and this two-dimensional subspace is termed the *branching space*. Necessarily, then, for an *N*-atom system, the degeneracy will be preserved at first-order in the space spanned by the remaining 3*N* – 8 internal coordinates, collectively denoted the *seam space*. Given the central role played by CIs in photochemistry and photophysics, the ability of ML models to quantitatively reproduce the branching and seam-space topographies is a problem previous works have not directly addressed.^8−15^ One approach to circumvent this issue is to instead learn quasi-diabatic potentials, which are expected to be smooth functions of nuclear coordinates.^16−18^ This procedure will, in general, however, require a (nonunique) choice as to how the surfaces will be diabatized and typically requires user-input and intuition beyond the computation of adiabatic electronic energies.

In this Letter, we demonstrate that accurate ML models of CI seams, including a correct description of the branching space, can, in fact, be constructed using just adiabatic electronic energies by forgoing the direct learning of PESs. Instead, we advocate for the learning of the nuclear coordinate-dependent coefficients of the characteristic polynomial (CP) of a simple decomposition of the potential matrix. This proposal follows directly from the previous work of Opalka and Domcke who used expansions of the CP coefficients in terms of permutationally invariant polynomials to fit models of intersecting adiabatic potentials.^19^ Unlike the adiabatic PESs, these quantities form smooth surfaces, even at points of CI. Furthermore, a simple mapping exists between the CP coefficients and the adiabatic PESs, making the recovery of the latter a trivial exercise. The efficacy of the proposed approach is demonstrated via the construction of kernel ridge regression (KRR) models of conical intersections for a number of small molecules, although the methodology adopted here will be transferable to other ML methods, e.g., the construction of neural network potentials.^20^

Let **R** denote the vector of 3*N* nuclear coordinates and **V**(**R**) the *n* × *n* nuclear coordinate-dependent adiabatic potential matrix with on-diagonal elements *V*_*ii*_(**R**) corresponding to the PESs *E*_*i*_(**R**) of interest. We begin with the decomposition of the potential matrix into an average energy and splitting matrix:
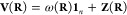
1where ω(**R**) = Tr **V**(**R**)/*n* denotes the average adiabatic energy, and the diagonal splitting matrix **Z**(**R**) has elements *Z*_*ij*_(**R**) = [*E*_*i*_(**R**) – *ω*(**R**)]δ_*ij*_. The average energy *ω*(**R**) is a smooth function of the nuclear coordinates, even at points of CI. To arrive at a smooth representation of the splitting contribution **Z**(**R**), Opalka and Domcke^19^ considered its characteristic polynomial:
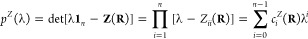
2where the CP coefficients *c*_*i*_^*Z*^(**R**) are given by
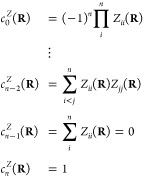
3

The CP coefficients are also smooth functions of the nuclear coordinates over any domain, including one that contains a CI seam. It is interesting to note that the characteristic polynomial of any given matrix is invariant with respect to similarity transformations of that matrix. Hence, the CP coefficients of the splitting matrix **Z** are invariant with respect to the choice of electronic basis. Thus, the same set of CP coefficients is generated in the adiabatic representation as for a diabatic representation. The same holds for the *ω*(**R**) term, which is the trace of the potential matrix, an operation whose result is also invariant to the choice of the electronic basis.

From the set of CP coefficients, {*c*_*i*_^*Z*^(**R**)}, and average energy, *ω*(**R**), the adiabatic potentials may easily be recovered as the eigenvalues of the following companion matrix:^19^


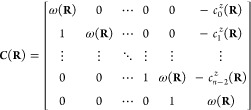
4The advantages of learning the set of functions {*ω*(**R**), *c*_*i*_^*Z*^(**R**): *i* = 0, ..., *n* – 2} instead of the PESs *E*_*i*_(**R**) are two-fold. First, these functions are extremely smooth, as illustrated in Figure 1 for a simple first-order model of a two-state CI. This, in turn, renders them highly amenable to machine learning, unlike the underlying adiabatic PESs, which exhibit discontinuous derivatives at a point of CI. Second, and somewhat remarkably, all of the branching space information is contained in the single CP coefficient *c*_*n*–2_^*Z*^(**R**), irrespective of the number of intersecting states. To see this, we first note that the following identity holds:
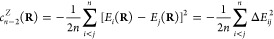
5The proof of this is somewhat nonobvious and is given in the Supporting Information. Now, since each squared energy difference Δ*E*_*ij*_^2^(**R**) is lifted to second-order at a point of CI with respect to at least one the branching space coordinates, and to fourth-order with respect to all the seam space coordinates, the Hessian
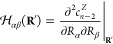
6when evaluated at a point of CI, **R**_CI_, has a column space corresponding to the branching space and a null space corresponding to the seam space. Thus, the orthogonalized branching (seam space) coordinates may be computed as the eigenvectors of  with nonzero (zero) eigenvalues.^21−23^ Further, as detailed below, the nonzero eigenpairs can be used to characterize the conical intersection topography. As such, in addition to being smooth, slowly varying functions, the CP coefficients provide a simple yet fundamental encapsulation of the branching space information, irrespective of the number of intersecting states.

**Figure 1:**
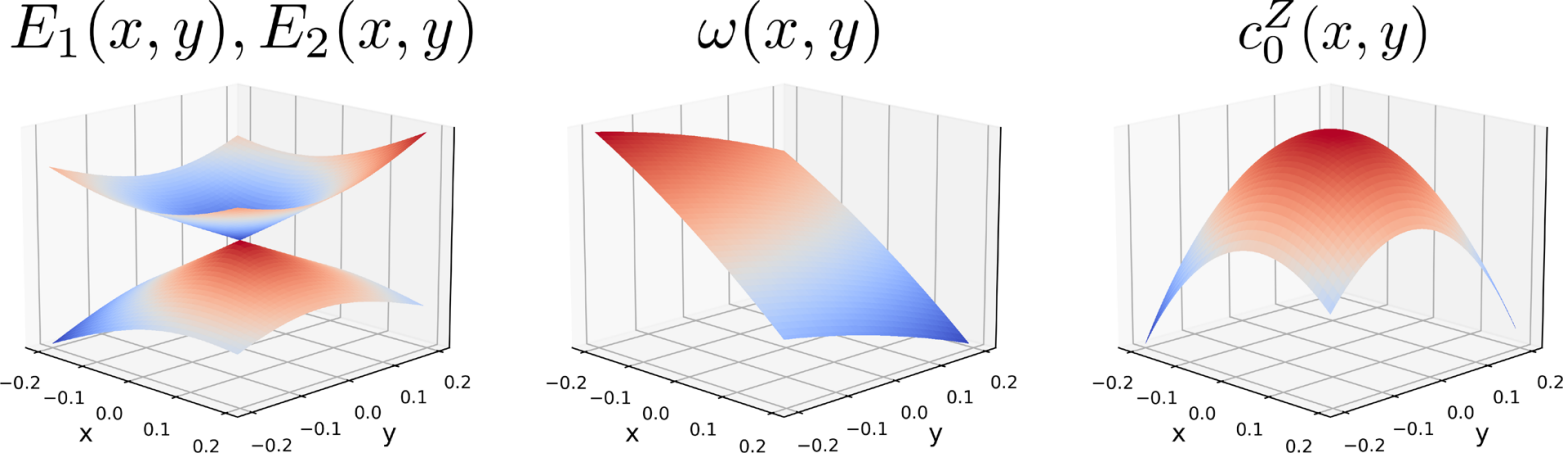
Adiabatic potential energy surfaces, *E*_1_(*x*, *y*) and *E*_2_(*x*, *y*); average energy, *ω*(*x*, *y*); and the single splitting matrix CP coefficient, *c*_0_^*Z*^(*x*,*y*); for a representative first-order CI model (eq 7) with parameters *g̃* = *h̃* = 0.5, *s*_*x*_ = 0.25, and *s*_*y*_ = 0.1. Unlike the adiabatic potential energy surfaces, *ω*(*x*, *y*) and *c*_0_^*Z*^(*x*,*y*) are smooth, differentiable functions even at a point of CI.

To show the advantages of constructing ML models of seams of CIs using the above-described CP formalism, KRR models were constructed for three prototypical CIs: (1) the symmetry-required (*E* ⊗ *e*) two-state CI between the two components of the *D*_1_ state of NH_3_^+^, (2) the accidental “twisted-pyramidalized” (TwPy) CI between the *S*_0_ and *S*_1_ states in ethylene, and (3) the symmetry-required (*T*_2_ ⊗ (*e* ⊕ *t*_2_)) three-state intersection between the components of the *D*_0_ state of CH_4_^+^. In addition, in order to highlight the shortcomings of the traditional, direct approach, KRR models of the adiabatic PESs themselves were computed for all three systems. In the following, we shall refer to these two sets of models as the “*ω*-CP” and “direct-energy” models.

In all cases, the radial basis function (RBF) kernel was used in conjunction with the Smoothed Overlap of Atomic Positions (SOAP) descriptor.^24,25^ In brief, the SOAP descriptor transforms the Cartesian nuclear coordinates into a power spectrum through a linear combination of a Gaussian-type orbital functions and spherical harmonics centered at each atomic site, which satisfies the desired symmetries and bijection of a molecular descriptor. More importantly, the SOAP descriptor allows for scaling to large molecules due to being independent of the molecule size.^26^ The parameters of the SOAP descriptor were separately optimized for each system using a genetic algorithm approach. The adiabatic energies that comprised the training sets were computed at the multireference configuration interaction (MRCI) level of theory, in which the reference space and orbital basis were generated from a minimum orbital complete active space self-consistent field (CASSCF) calculation using the *COLUMBUS* electronic structure package.^27^ The nuclear structures were generated via Latin hypercube sampling about each MECI geometry. Further details of these calculations are given in the Supporting Information.

We first consider the quality of the *ω*-CP and direct-energy KRR models as judged by the mean absolute errors (MAEs) in the vicinity of the MECI geometries as a function of training set size. These are shown in Figure 2. In all cases, subchemical accuracy in the learned PESs is attained with fewer than 300 training points. We note, however, that the PESs obtained from the *ω*-CP KRR models are consistently more accurate for a given training set size than the corresponding direct-energy models. The ability of the direct-energy models to accurately describe, on average, the adiabatic PESs in a subspace containing a CI, however, belies a larger failing. Namely, an inability to correctly describe the branching spaces in terms of both direction and dimensionality. To see this, we consider the eigenvalues of the *c*_*n*–2_^*Z*^(**R**) Hessian  (eq 6) evaluated at the MECI points using both the *ω*-CP and direct-energy models. These are shown in Figure 3 alongside the *ab initio* MRCI values. The number of nonzero eigenvalues of  should equal the dimensionality of the branching space: two for a two-state CI and five for a three-state CI. In all cases, the *ω*-CP models quantitatively reproduce the *ab initio* eigenvalues. On the other hand, the direct-energy models fail rather badly: for the NH_3_^+^ and CH_4_^+^ models, the dimensionality of the predicted branching space is too high. For the C_2_H_4_ model, only two significantly nonzero eigenvalues are furnished by the direct-energy model. However, the values of these are significantly underestimated, which, as we discuss below, is related to an incorrect description of the CI topography.

**Figure 2:**
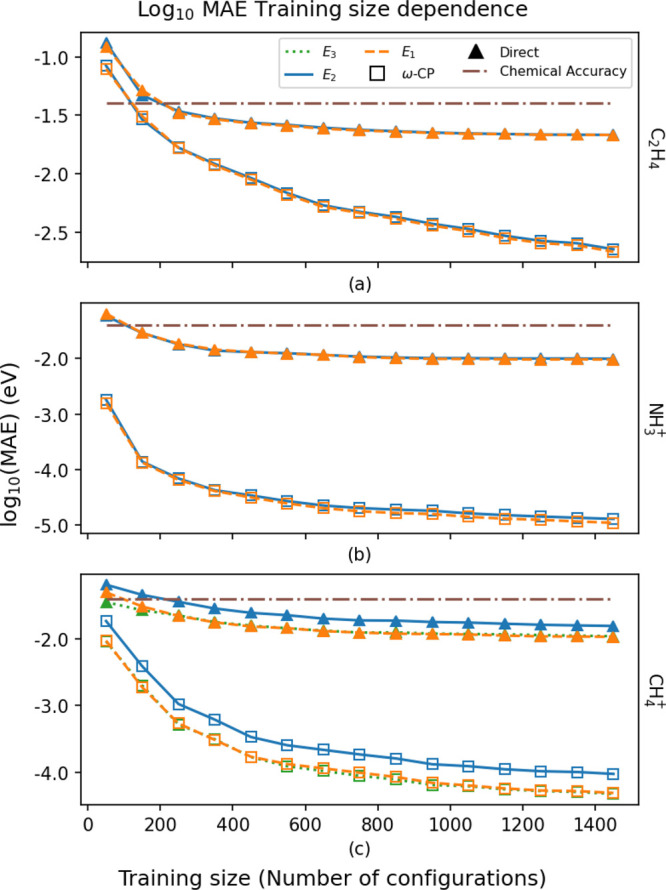
The log of mean absolute error as a function of training set size, in terms of the number of nuclear configurations, when fitting the characteristic polynomial parameters (*ω*-CP) or the adiabatic energies (Direct).

**Figure 3:**
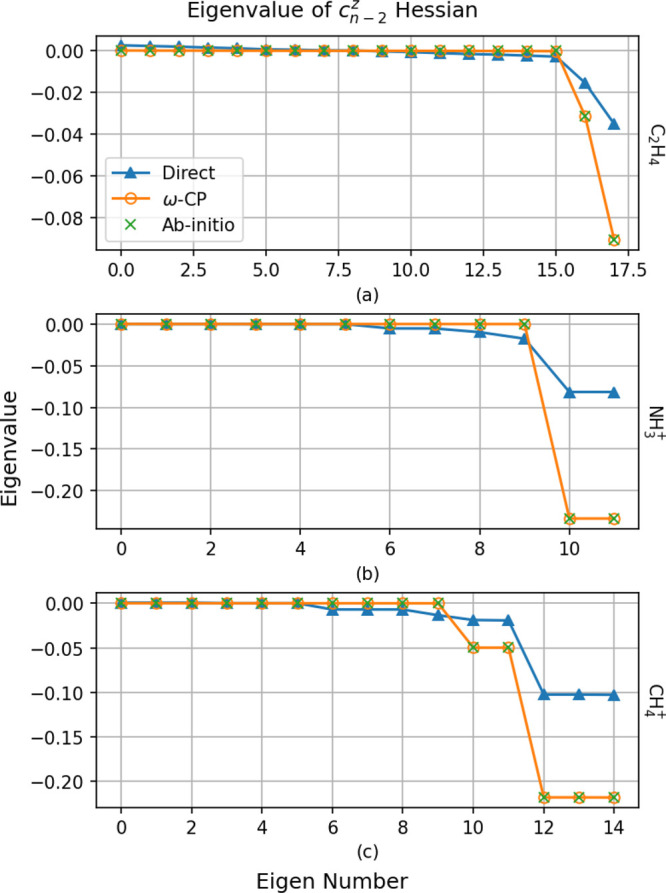
Eigenvalues of the *c*_*n*–2_^*z*^ Hessian matrix, as computed from *ab initio* data and using the *ω*-CP and direct energy fit model potentials. The number of nonzero eigenvalues corresponds to the dimension of the branching space.

Having established the ability of the *ω*-CP models to correctly recover the dimensionality of the branching space, we now consider their ability to describe the CI topography, that is, the parameters describing the tilt and pitch of the cone. Here, we restrict ourselves to the two-state CI case, which is described to first-order within the branching space by following model potential:^28,29^
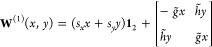
7Here, *g̃* and *h̃* are the norms of the orthogonalized gradient difference and derivative coupling vectors, respectively,
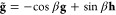
8
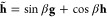
9
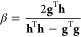
10where **g** and **h** denote the nascent gradient difference and derivative coupling vectors. Together, the parameters g̃ and h̃ describe the asymmetry in the pitch of the cone. The parameters
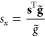
11and
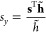
12
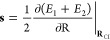
13describe the tilt of the principal axis of the CI cone. For the *ω*-CP models, all of the first-order parameters are trivial to compute. The branching space coordinates *x* and *y* correspond to the column space of the Hessian  of *c*_*n*–2_^*Z*^(**R**). The corresponding nonzero eigenvalues take values of −2*g̃*^2^ and −2*h̃*^2^,^21−23^ and the tilt parameters *s*_*x*_ and *s*_*y*_ are obtained using the relation
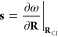
14The values of the first-order parameters computed using the *ω*-CP and direct-energy models, as well as the angles *θ*_*x*,*y*_ between the model and *ab initio* branching space vectors **x** and **y**, are shown in Table 1 for both two-state models. These results are derived from models fit to 3000 *ab initio* points. The *ω*-CP models are found to quantitatively reproduce the CI topography in both cases, with branching vector angles *θ*_*x*,*y*_ of less than 0.5° and parameters *g̃*, *h̃*, and *s*_*x*,*y*_ in almost perfect agreement with the *ab initio* values. In fact, the CP coefficients can reproduce the branching space with very modest training set sizes, as shown in Table S7 in the SI, achieving reasonable accuracy with training set sizes as small as 500 *ab initio* points. The direct-energy models, on the other hand, fare less well, with maximum branching vector angles *θ*_*x*,*y*_ of 8.0° and parameters *g̃*, *h̃*, and *s*_*x*,*y*_ that fail to correctly describe the CI topography, even with large training sets containing many thousands of points.

**Table 1:** Comparison of the Branching Space Parameters Determined from *ab Initio* Data and Using the Surrogate Potentials^a^

		θ_*x*_	θ_*y*_	*g̃*	*h̃*	*s*_*x*_	*s*_*y*_
C_2_H_4_	*ab initio*	0.0	0.0	0.213	0.125	–0.130	–0.046
	ω-CP	0.0	0.3	0.214	0.126	–0.130	–0.046
	direct-energy	3.7	8.0	0.130	0.084	0.128	–0.050
NH_3_^+^	*ab initio*	0.0	0.0	0.342	0.342	0.000	0.000
	ω-CP	0.3	0.3	0.342	0.342	0.000	0.000
	direct-energy	5.3	5.3	0.202	0.202	0.000	0.001

a Angles are given in units of degrees. All other values are given in units of *E*_h_/Å. See text for the formal definition of the parameters.

Next, we show in Figure 4 both sets of model potentials for the two-state systems along the two *ab initio* branching space coordinates, *x* and *y*. Two important aspects are immediately obvious. First, the direct-energy models actually yield avoided crossings, not CIs. On the other hand, the *ω*-CP models correctly reproduce the intersection of the PESs. Second, and perhaps more surprisingly, the *ω*-CP models are able to accurately extrapolate the PESs to geometries outside of the training sets used to construct them. To see this, we first refer to Figure 5, where the model branching space cuts are extended to large displacements. Both *ω*-CP models remain accurate out to displacements of *x* = 0.4 and *y* = 0.4, which correspond to geometries outside the span of the training sets. This can be discerned from Figures S3 and S4, in which we show, respectively, a superposition of the training set geometries, and the geometries corresponding to *x* = 0.4. Somewhat remarkably, for ethylene, this geometry corresponds to ethylidene, a structural isomer different from the geometries present in the training set. Similarly, the ammonia geometry at *x* = 0.4 corresponds to a near-dissociated N–H bond which, again, is not represented in the training set. The improved ability of the *ω*-CP models to extrapolate is a direct result of the significantly longer length scales on which the surfaces *ω*(**R**) and *c*_*i*_^*Z*^(**R**) vary compared with the PESs *E*_*i*_(**R**). This, in turn, results in large kernel length scales and an increased distance from which knowledge may be transferred from the training to prediction points. On the other hand, the rapidly varying adiabatic PESs result in direct-energy KRR models that rapidly lose all predictive power when moving away from elements of the training set.

**Figure 4:**
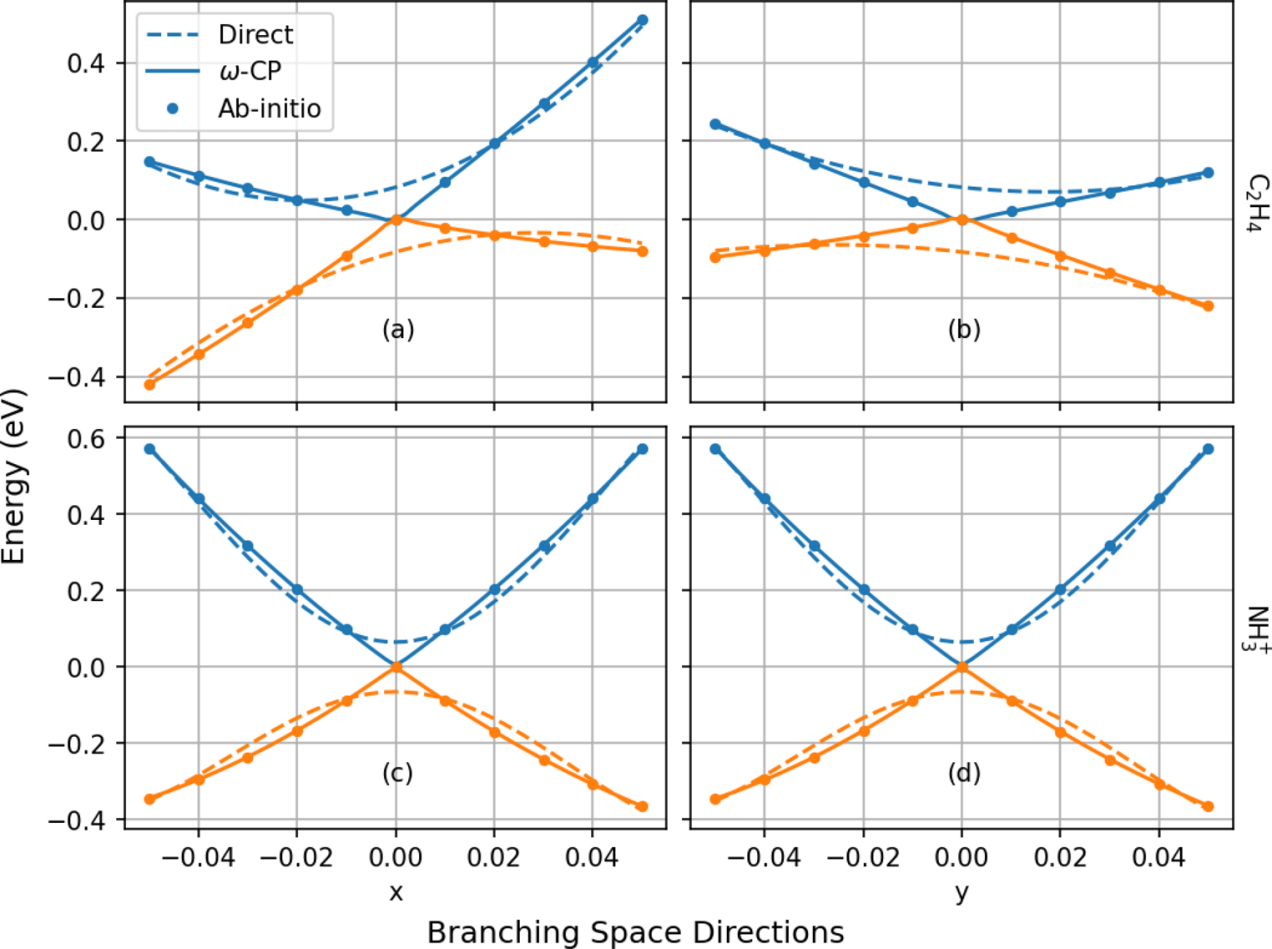
Adiabatic potentials in the immediate vicinity of a minimum energy conical intersection as determined from *ab initio* computations (isolated points) and the *ω*-CP and direct energy surrogate potentials. The latter fails to capture the degeneracy and instead shows an avoided crossing.

**Figure 5:**
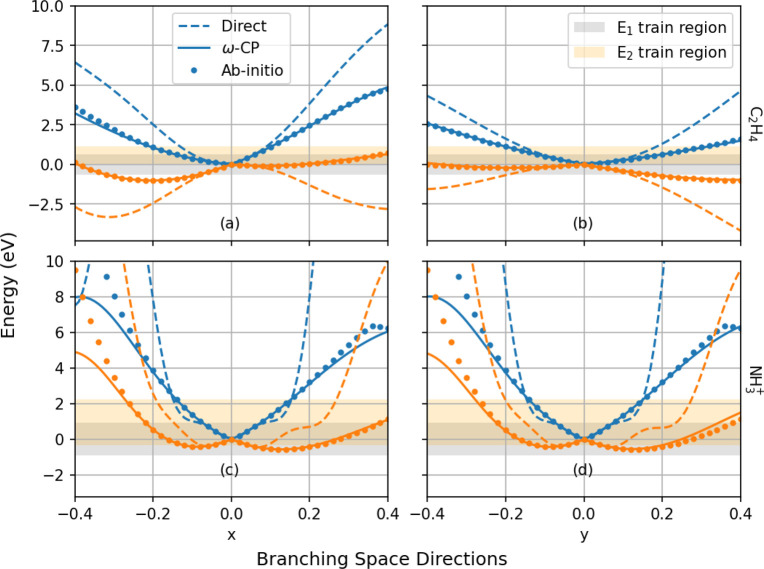
Adiabatic potentials over an extended region of coordinate space along the (rectilinear) branching space directions *x* and *y*, for the two-state examples. The shaded regions show the energetic extent of the fit set used to generate the direct and *ω*-CP models. The *ω*-CP models exhibit impressive fidelity to the *ab initio* surfaces over an energy range significantly exceeding that of the fit data.

It remains to comment on the choice of kernel. The RBF kernel used here is a simple “default” choice in many kernel regression based methods. Belonging to the *C*^*∞*^ differentiability class, it performs well for the smooth *ω*(**R**) and *c*_*i*_^*Z*^(**R**) functions, the main focus of this work. However, for the direct learning of the PESs *E*_*i*_(**R**), other kernel choices might give improved results. An obvious choice here is the Matérn-1/2 kernel, which belongs to the same differentiability class (*C*^0^) as a set of intersecting adiabatic PESs. However, even using such a kernel, poor accuracy of the direct-energy KRR models was attained. This is demonstrated in Figures S2 and S3, where the direct-energy Matérn-1/2 kernel model PESs are plotted along the branching space coordinates for ethylene. Even with this nondifferentiable kernel choice, the model PESs still form an avoided crossing instead of a CI. Yet more complicated, and potentially better performing, kernels could be conceived of, e.g., additive kernels designed to capture strong subdimensional interactions.^30^ However, we believe that a strength of our proposed approach is that the complex intersecting PES structure can be transformed into functions that can be well described by a simple kernel, as exemplified by the RBF kernel.

Finally, we compare the proposed *ω*-CP approach to learning seams of CI to alternative approaches. An obvious alternative would be to learn quasi-diabatic potential matrices,^16,31,32^ the elements of which are also smooth functions of the nuclear coordinates. Indeed, this would be an advantageous approach if nonadiabatic couplings are also needed. If this is not the case, however, then the *ω*-CP approach seems to be advantageous for three main reasons. First, quasi-diabatic potentials are somewhat ill-defined, a result of the nonexistence of strictly diabatic states for polyatomic molecules.^33^ The functions *ω*(**R**) and *c*_*i*_^*Z*^(**R**), on the other hand, form a fundamental representation of the potential matrix, as result of being invariant to unitary transformations of the choice of electronic basis. Second, and related to the prior point, *ω*(**R**) and *c*_*i*_^*Z*^(**R**) can be trivially computed from the adiabatic potentials *E*_*i*_(**R**), which are directly furnished from quantum chemistry calculations. This stands in contrast to quasi-diabatic potentials, for which a subsequent adiabatic-to-diabatic transformation must be applied, typically requiring either the additional calculation of derivative couplings or electronic wave function overlaps. This may be overcome by switching to a diabatization by *ansatz*, neural network-based implementations of which have recently been reported.^34−36^ However, such an approach requires additional, nonuniquely defined user-defined constraints to be imposed on the model quasi-diabatic potentials. Last, the set {*ω*(**R**), *c*_*i*_^*Z*^(**R**): *i* = 0, ..., *n*–2} contains only *n* quantities to be learned, while the quasi-diabatic potential matrix is composed of *n*(*n* + 1)/2 symmetry-unique elements. Thus, the *ω*-CP formalism provides a more compact representation of a set of *n* adiabatic PESs.

To conclude, we have demonstrated the ability to construct quantitatively accurate ML models of seams of CI via indirect learning of the involved adiabatic PESs based on a CP formalism. The advocated *ω*-CP approach yields a correct description of the branching and seam spaces, a feat that is harder to achieve for models based on the direct learning of adiabatic PESs. Furthermore, it is found that a single CP coefficient, *c*_*n*–2_^*Z*^(**R**), contains all branching space information, irrespective of the number of intersecting states. In terms of practical application, the *ω*-CP formalism will be useful in a number of situations. For example, in MECI optimization using quantum chemistry methods for which analytical gradients are not available; in such cases, *ω*-CP based surrogate potentials may straightforwardly be utilized. Additionally, the use of ML adiabatic PESs and nonadiabatic couplings in excited-state dynamics simulations is starting to gain traction.^37,38^ Here, there seems to be no reason to continue using directly learned adiabatic PESs, given the unambiguous advantages of the indirect *ω*-CP approach. We thus anticipate that the results presented here shall be of great use in directing future developments in this nascent, yet important, field of work.

## Supporting Information Available

Additional information on the generation of the training sets, KRR hyperparameter optimization, choice of kernel, proof of eq 5, and the ability of the ω-CP models to extrapolate (PDF)
Transparent Peer Review report available (PDF)
