# Supplementary material for: Machine Learning Seams of Conical Intersection: A Characteristic Polynomial Approach
Source: J Phys Chem Lett. 2023 Aug 24;14(35):7780–6. doi: 10.1021/acs.jpclett.3c01649 (PMC10494228; doi:10.1021/acs.jpclett.3c01649)
Supplement: Supplementary file 1 — jz3c01649_si_001.pdf [file jz3c01649_si_001.pdf]

Supplementary Information for Machine Learning Seams of Conical Intersection: A Characteristic Polynomial Approach

Tzu Yu Wang,† Simon P. Neville,*,‡ and Michael S. Schuurman*,‡,†

†*Department of Chemistry and Biomolecular Sciences, University of Ottawa, Ottawa, Canada, K1N 6N5*

‡*National Research Council Canada, 100 Sussex Dr., Ottawa, Canada, K1A 0R6*

E-mail: simon.neville@nrc-cnrc.gc.ca; michael.schuurman@uottawa.ca

Training sets

Sampling

Training sets were generated by Latin hypercube (LHC) sampling about each minimum energy conical intersection (MECI) geometry. In all cases, the LHC sampling was performed in terms of Cartesian coordinates with bounds of $\pm 0.05\mathring{A}$ for each degree of freedom. 10000 geometries were generated per molecule, which were subsequently split into training and test sets, as further detailed below.

Quantum chemistry calculations

Ab initio energies were computed at the multi-reference configuration interaction (MRCI) level of theory, from which average energies, ω, and characteristic polynomial coefficients, c_i^Z, were generated. The MECI geometries about which the adiabatic energies were sampled were computed at the same (MRCI) level of theory. In these calculations, complete active space self-consistent field (CASSCF) calculations were used to generate the molecular orbital (MO) bases and to provide the MRCI reference spaces. CAS(2,2), CAS(5,3), and CAS(5,3) reference spaces were used for C_2H_4, NH_3^+, and CH_4^+, respectively. In all CASSCF calculations, state averaging was performed over the three lowest-lying states. MRCI configurations were then generated by allowing all single excitations out of the reference space. The cc-pVDZ basis was used in all calculations. The CASSCF and MRCI calculations were performed using the Columbus set of programs.[1]

MECI geometries

Below, we give the Cartesian coordinates of the MRCI-optimized MECI geometries used.

Table 1: NH_3^+

4

N	0.0000000	0.0000000	0.1170240
H	0.0000000	0.9353610	-0.2730560
H	0.8100460	-0.4676800	-0.2730560
H	-0.8100460	-0.4676800	-0.2730560

Table 2: C_2H_4

6

C	-0.642746	0.019430	-0.000001
C	0.763577	-0.099359	-0.117167
H	-1.200659	0.962608	0.015890
H	-1.250756	-0.852848	0.246114
H	0.646531	0.597069	0.826375
H	1.293609	0.672245	-0.693380

Table 3: CH_4^+

5

C	0.0000000	0.0000000	0.0000000
H	0.0000000	-0.8899253	-0.6292722
H	-0.8899255	0.0000000	0.6292720
H	0.0000000	0.8899253	-0.6292722
H	0.8899255	0.0000000	0.6292720

Kernel ridge regression calculations

SOAP descriptor

To efficiently predict structure-property relations with machine learning (ML) we need to encode the structural information in an ML-friendly way. The main requirements of any descriptor can be summarized as follow:

1. Invariant to spatial translations
2. Invariant to rotation of the coordinate system
3. Invariant with permutations of atomic indices
4. Complete and unique: the structure and descriptor representation has a bijective mapping and each property corresponds to a unique descriptor
5. Continuous: 'small' changes in the structure result in a 'small' change in the descriptor
6. Non-redundant and computationally cheap to construct

The smoothed overlap of atomic positions (SOAP) descriptor[2] satisfies all these criteria and is the descriptor of choice in this work. The SOAP descriptor encodes local atomic environments through a sum of Gaussian-type densities placed at a number of local centres, and subsequently expanded in terms of Gaussian radial and spherical harmonic angular basis functions. These local environments are expressed as power spectrum and can be placed arbitrarily in real space, but they are often, and in this work, placed on each atomic centre in the molecule. These power spectra satisfy the above requirements of a descriptor, the proof and derivations can be found in (Bartok 2013).[2] A partial power spectrum placed on a single centre is given by

$$p_{nn'l}^{Z_1 Z_2} = \pi \sqrt{\frac{8}{2l+1}} \sum c_{nlm}^{Z_1} c_{n'lm}^{Z_2}, \tag{S1}$$

where n is the indexes radial basis indices, and l and m index the angular spherical harmonic basis functions. The coefficient c^Z_{nlm} is defined through the following inner product:

$$c^Z_{nlm} = \iiint_V dV g_n(r) Y_{lm}(\theta, \phi) \rho^Z(r), \tag{S2}$$

$$\rho^Z(r) = \sum_i^{|Z_i|} e^{-\frac{1}{2\sigma^2 |r - R_i|^2}}, \tag{S3}$$

$$g_{nl}(r) = \sum_n^{n_{max}} \beta_{nn'l} r^l e^{-\alpha_{nl} r^2}, \tag{S4}$$

where ρ^Z in Equation S3 is a species-dependent pseudo-atomic density built from a Gaussian controlled by the width parameter σ, and the Y_{lm} are spherical harmonics. The decay parameter α_n in the Gaussian radial function g_n is chosen such that each term in g_n decays to a threshold value of 10^{-3} at a cutoff radius r_{cut}. A full power spectrum that represents a local environment is taken as the concatenation of the partial power spectrum $p^{Z_1 Z_2}_{nn'l}$ for all unique pairs of atomic species. A global SOAP descriptor $\boldsymbol{P}$ with elements

$$P_I = P^{Z_1 Z_2}_{nn'l} \sim \sum_m \left(\frac{1}{n} \sum_i c^{i, Z_1}_{nlm} \right) \left(\frac{1}{n} \sum_i c^{i, Z_2}_{nlm} \right) \tag{S5}$$

is then built from a sum over the local power spectra centered at each site. The size of the SOAP descriptor $\boldsymbol{P}$ therefore does not depend directly on the size of the molecule, but rather the dimension of the angular basis $\{Y_{lm}\}$ and number of atomic species included in Equation S1. The length of the resulting power spectrum can be calculated as

$$L = \frac{1}{2} n_{max} S_n (n_{max} S_n + 1)(l_{max} + 1), \tag{S6}$$

where S_n is the number of atomic species included.

The SOAP descriptor is specified by four parameters: r_{cut}, n_{max}, l_{max}, σ. The parameters n_{max} and l_{max} specifies the angular expansion of the spherical harmonic in Equation S2, σ

controls the width of the Gaussian-type pseudo-density in Equation S3, and r_{cut} implicitly changes the decay parameter in Equation S4, which controls the maximum reach of the radial basis. Computations of the SOAP descriptor were carried out using the DScribe Python package.[3,4]

Kernel functions

All results presented in the main text were computed using the anisotropic Radial Basis Function (RBF) (i.e., squared exponential) kernel,

$$k_{RBF}(\boldsymbol{P}_i, \boldsymbol{P}_j) = \exp\left(-\frac{d(\boldsymbol{P}_i, \boldsymbol{P}_j)^2}{2l^2}\right), \tag{S7}$$

where $d(\boldsymbol{P}_i, \boldsymbol{P}_j)$ is the Euclidean distance between SOAP feature vectors $\boldsymbol{P}_i$ and $\boldsymbol{P}_j$. The RBF kernel function is infinitely differentiable and well suited to the description of the smooth $\omega(\boldsymbol{R})$ and $c_i^Z(\boldsymbol{R})$ functions. On the other hand, the non-differentiable adiabatic PESs $E_i(\boldsymbol{R})$ forming a conical intersection could reasonably be assumed to be better described using a non-differentiable kernel. To investigate this, additional calculations were performed using the Matérn-1/2 kernel

$$k_{1/2}(\boldsymbol{P}_i, \boldsymbol{P}_j) = \exp\left(-\frac{d(\boldsymbol{P}_i, \boldsymbol{P}_j)}{l^2}\right). \tag{S8}$$

Optimization of the SOAP and kernel hyperparameters

The SOAP parameters r_{cut}, n_{max}, l_{max}, σ and the kernel hyperparameter length scale l are optimized simultaneously using a Genetic Algorithm (GA) search. A GA was used as we require the simultaneous optimization of a mixed set of continuous and discrete variables, which makes gradient-based optimization methods difficult. The GA search algorithm follows a generic procedure of creating a population, selecting top candidates based on chosen metric and random selection, then allowing combination and random mutation which form a new

set of population. The process is repeated until an optimal solution is found. First, an initial population is generated by randomly sampling from the bounded set of parameter values:

- $r_{cut} \epsilon [1, 9] \subset \mathbb{R}$
- $n_{max} \epsilon [2, 9] \subset \mathbb{Z}^+$
- $l_{max} \epsilon [2, 9] \subset \mathbb{Z}^+$
- $\sigma \epsilon [10^{-3}, 10^{-1}] \subset \mathbb{R}$
- $l_{kernel} \epsilon [10^{-5}, 10^{-1}] \subset \mathbb{R}$

Once a population (paramter set) is generated, a metric is used to score each individual (sets) in the population, here taken as the root mean squared displacement (RMSD) of KRR model predictions relative to a test set. The training/test sets were generated by randomly splitting the data set into 15%/85% subsets. Once scored by their RMSDs, survival of the fittest is applied, and from the remaining survivors, crossover and mutation operations are performed to generate a new population (set of paramters) of the same length, and the whole process is repeated until convergence. Simulated Binary Crossover (SBX) and Polynomial Mutation (PM) algorithms were used for the crossover and mutation procedure, respectively. The GA search is carried out with pymoo[5]

Effect of the choice of kernel

We here consider the effect of using a non-differentiable kernel on the ability of the direct-energy KRR models to correctly reproduce a conical intersection. For this purpose, we show in Figures S1 and S2 the direct-energy model PESs for the ethylene example obtained using the Matérn-1/2 kernel. As can clearly be seen, even when using a kernel belonging to the same differentiability class as the adiabatic PESs, the direct-energy models still fail to reproduce the intersection of the PESs, instead furnishing an avoided crossing.

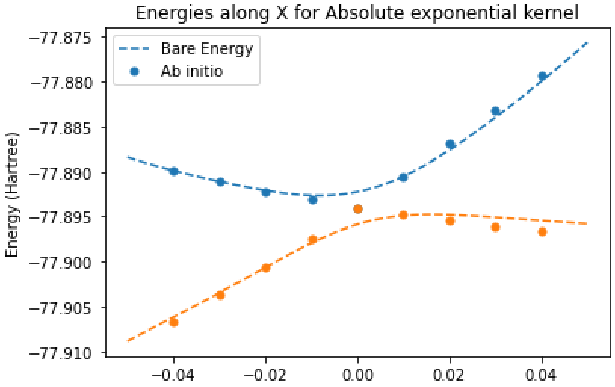

Figure S1: *Ab initio* and direct-energy Matérn-1/2 KRR model PESs along the x branching space coordinate from the ethylene TwPy MECI.

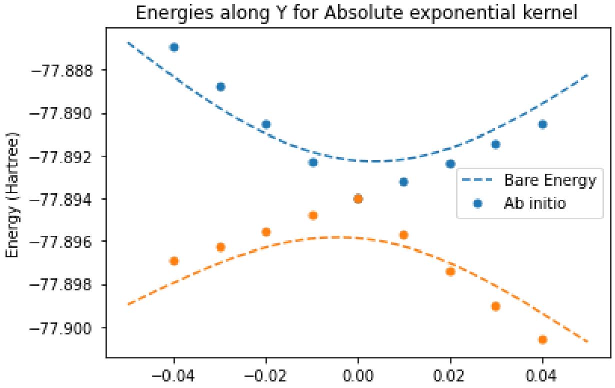

Figure S2: *Ab initio* and direct-energy Matérn-1/2 KRR model PESs along the y branching space coordinate from the ethylene TwPy MECI.

Proof of Equation 5

We start by expanding out the right hand side of Equation 5:

$$\sum_{i<j}^{n}(E_i - E_j)^2 = \sum_{i<j}^{n}\left[(E_i - \omega) - (E_j - \omega)\right]^2 = \sum_{i<j}^{n}(Z_{ii} - Z_{jj})^2 \tag{S9}$$

Expanding out Equation S9 and multiplying out the summation we obtain

$$\sum_{i<j}^{n}(E_i - E_j)^2 = \sum_{i<j}^{n}(Z_{ii}^2 + Z_{jj}^2) - 2\sum_{i<j}^{n} Z_{ii}Z_{jj} \tag{S10}$$

Taking the first term on the right-hand side of Equation S10, expanding it, and simplifying

the double ordered summation to a single summation, we obtain

$$\sum_{i<j}^{n}(Z_{ii}^2 + Z_{jj}^2) = (n-1)\sum_{i}^{n} Z_{ii}^2 \tag{S11}$$

We now make use of the trace property of a Kronecker product

$$Tr(\boldsymbol{Z} \otimes \boldsymbol{Z}) = Tr(\boldsymbol{Z})Tr(\boldsymbol{Z}) = 0, \tag{S12}$$

where the last equality in Equation S12 holds because the diagonal splitting matrix $\boldsymbol{Z}$ is by construction traceless. We now note that the trance tensor product of an $n \times n$ diagonal matrix $\boldsymbol{Z}$ with itself can be written as a sum of the form

$$Tr(\boldsymbol{Z} \otimes \boldsymbol{Z}) = \sum_{i}^{n} Z_{ii}^2 + 2\sum_{i<j}^{n} Z_{ii}Z_{jj} \tag{S13}$$

Setting Equation S13 to zero from the result of Equation S12 gives

$$\sum_{i}^{n} Z_{ii}^2 = -2\sum_{i<j}^{n} Z_{ii}Z_{jj} \tag{S14}$$

Substituting Equation S14 into the right-hand side of Equation S11, we obtain the relationship

$$\sum_{i<j}^{n}(Z_{ii}^2 + Z_{jj}^2) = -2(n-1)\sum_{i<j}^{n} Z_{ii}Z_{jj} \tag{S15}$$

Equation S15 can now be substituted into Equation S10. and after simplifying we get the result

$$\sum_{i<j}^{n}(E_i - E_j)^2 = -2n\sum_{i<j}^{n} Z_{ii}Z_{jj} \tag{S16}$$

The sum term on the right-hand side of Equation S16 is precisely the definition of the c_{n-2}^{Z} CP coefficient,

$$c_{n-2}^Z = \sum_{i<j}^{n} Z_{ii} Z_{jj}. \tag{S17}$$

Thus, the desired result is obtained:

$$c_{n-2}^Z = -\frac{1}{2n} \sum_{i<j}^{n} (E_i - E_j)^2 \tag{S18}$$

Ability of the ω-CP models to extrapolate

We here consider the ability of the ω-CP KRR models to extrapolate well beyond the sub-volume of nuclear configuration space spanned by the geometries present in the training sets used to construct them. For reference, a superposition of all training set geometries for the ethylene and ammonia cation examples are shown in Figure S3. The tight nature of the LHC sampling used is clearly seen. Even so, both models are found to remain accurate out to relatively large displacements along the branching space coordinates x and y. To demonstrate this, we consider the geometries corresponding to $\boldsymbol{R}_{CI} + 0.4x$ and $\boldsymbol{R}_{CI} + 0.4y$, points at which the ω-CP model PESs are still accurate for both systems (as can be discerned from Figure 5 in the main text). These geometries are shown in Figure S4 alongside the MECI geometries $\boldsymbol{R}_{CI}$. As can be seen, these geometries lie outside of the span of the set of displacements of the training set geometries from the MECI geometries. Especially stark is the $\boldsymbol{R}_{CI} + 0.4x$ ethylene geometry, which corresponds to a different structural isomer to the geometries in the training set. Also notable is the ability of the ammonia cation ω-CP model to accurately describe the PESs at near-dissociated geometries. We thus conclude that, in stark contrast to the direct-energy KRR models, the ω-CP KRR models are able to extrapolate rather far beyond their training set data. This, in turn, is a result of the long length scales l on which the extremely smooth $\omega(\boldsymbol{R})$ and $c_i^Z(\boldsymbol{R})$ surfaces vary.

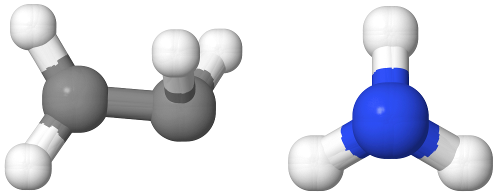

Figure S3: Superposition of the 10000 training set geometries used in the construction of each of the ethylene and ammonia models.

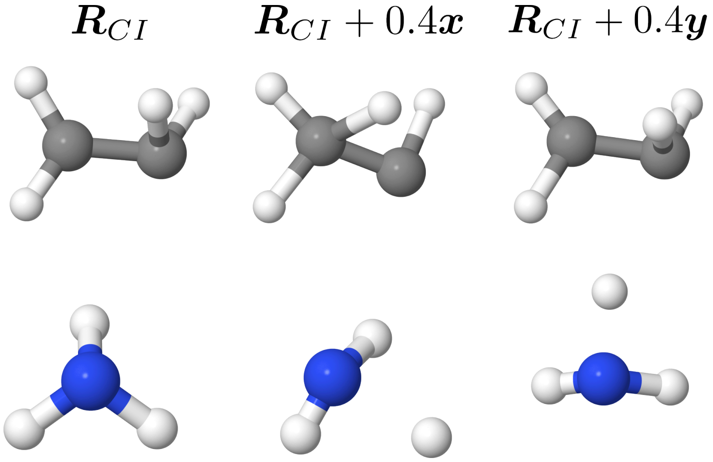

Figure S4: MECI geometries, $\boldsymbol{R}_{CI}$, and displacements of length 0.4 $\AA$ along the branching space directions x and y.

ML computational detail

Below are tables for C2H4, NH3 and CH4 containing all relevant ML parameters. r, n, l and σ are SOAP parameters and ls corresponds to the an-isotropic RBF kernel lengthscale.

Table 4: Relevant parameter for C2H4 KRR model

C_2H_4	r	n	l	σ	ls
ω	6.7891	7	8	0.100	0.1871
C_0^z	8.3599	6	8	0.100	0.1194
E_1	7.1588	5	7	0.078	0.8428
E_2	6.0168	4	7	0.099	0.6657

Table 5: Relevant parameter for NH3+ KRR model

NH_3^+	r	n	l	σ	ls
ω	8.3186	5	4	0.0026	0.0576
C_0^z	4.5722	6	4	0.0760	0.0951
E_1	2.8059	7	9	0.0060	0.4758
E_2	2.5946	4	7	0.0145	0.2089

Table 6: Relevant parameter for CH4+ KRR model

CH_4^+	r	n	l	σ	ls
ω	8.7078	9	5	0.0664	0.0908
C_0^z	6.3138	7	8	0.0880	0.0568
C_1^z	4.2123	5	8	0.0813	0.0769
E_1	2.0756	4	9	0.0129	0.0836
E_2	2.0326	4	9	0.0139	0.0724
E_3	2.0481	5	9	0.0072	0.1025

Dependence of branching space topography parameters with reduced training size

Table 7 shows a comparison between the ω-cp learned branching space parameters with the ab-initio values at reduced training sizes. Parameters shown in Table 1 of main text uses N=3000.

Table 7: Dependence of branching space topography parameters with respect to training set size (N). N=200, N=500 and N=1500 is shown. Reasonable accuracy is obtained at N=500 and approaches ab-initio values at N=1500

C_2H_4		θ_x	θ_y	$\tilde{g}$	$\tilde{h}$	s_x	s_y
Ab initio		0.0	0.0	0.213	0.125	-0.130	-0.046
N=200	ω-cp	2.4	8.9	0.216	0.125	-0.130	-0.041
N=500	ω-cp	0.9	1.6	0.216	0.127	-0.130	-0.045
N=1500	ω-cp	0.3	0.5	0.214	0.126	-0.130	-0.046

References

(1) Lischka, H.; Shepard, R.; Shavitt, I.; Pitzer, R. M.; Dallos, M.; Müller, T.; Szalay, P. G.; Brown, F. B.; Ahlrichs, R.; Böhm, H. J. et al. COLUMBUS, an ab initio electronic structure program, release 7.0. 2012.

(2) Bartók, A. P.; Kondor, R.; Csányi, G. On representing chemical environments. *Phys. Rev. B* **2013**, *87*, 184115.

(3) Himanen, L.; Jäger, M. O. J.; Morooka, E. V.; Federici Canova, F.; Ranawat, Y. S.; Gao, D. Z.; Rinke, P.; Foster, A. S. DScribe: Library of descriptors for machine learning in materials science. *Computer Physics Communications* **2020**, *247*, 106949.

(4) Laakso, J.; Himanen, L.; Homm, H.; Morooka, E. V.; Jäger, M. O. J.; Todorović, M.; Rinke, P. Updates to the DScribe Library: New Descriptors and Derivatives. 2023.

(5) Blank, J.; Deb, K. pymoo: Multi-Objective Optimization in Python. *IEEE Access* **2020**, *8*, 89497–89509.
