# Supplementary material for: Machine Learning Seams of Conical Intersection: A Characteristic Polynomial Approach
Source: J Phys Chem Lett. 2023 Aug 24;14(35):7780–6. doi: 10.1021/acs.jpclett.3c01649 (PMC10494228; doi:10.1021/acs.jpclett.3c01649)
Supplement: Supplementary file 2 — jz3c01649_si_002.pdf [file jz3c01649_si_002.pdf]

jz-2023-01649z.R1

Name: Peer Review Information for "Machine Learning Seams of Conical Intersection: A Characteristic Polynomial Approach"

First Round of Reviewer Comments

Reviewer: 1

Comments to the Author

The manuscript dealt with smoothly fitting seam surface of conical intersections between two adiabatic potential energy surfaces by decomposing potential matrix into the coordinate-dependent coefficients of the characteristic polynomial. The method is interesting as sort of diabatization of adiabatic potential energy surfaces. The machine learning part is just routine work. Thus, I do not feel that the manuscript is suitable for publication on The Journal of Physical Chemistry Letters.

Reviewer: 2

Comments to the Author

This paper proposed a convenient way to build machine learning (ML) potential energy surfaces (PES) coupled with conical intersections (CI) with coordinate-dependent characteristic polynomial (CP). As addressed for example systems, both the adiabatic energies and PES topology in vicinity of CI could be correctly reproduced with CP model compared with conventional treatment. It seems that, with CP build PES, obtain reasonably statistical properties, i.e. quantum yields and lifetimes, upon propagating nonadiabatic molecular dynamics trajectories is desirable. However, to verify the validity and efficiency of CP model, detailed comparison with previously reported ML-based PES construction algorithms and more elaborate tests should be presented before this paper be published.

1. The model system in current work only covers CI region. However, as the future of CP model is build PES for nonadiabatic molecular dynamics simulation, the moderate example would naturally covers an entire relaxation pathway, i.e. double bond twisting of ethylene. The difficulty for fitting PESs coupled with CIs not only the CI region itself but also the switching area toward CI. Moreover, the authors claimed that the CP based PESs could extrapolated to area faraway from training set geometries, it is quite interesting that whether the CP model could build PES with sparser ab initio geometry grids?

2. In current paper, only the fitted adiabatic potentials were presented, the performance of energy gradients with CP based PESs are not tested, which is of crucial importance to ensure trajectory propagation on PESs follow reasonable reaction pathways.

3. In Page 14, the authors have addressed one of the advantage of CP model compared with diabatic PES is free of additional calculations beyond potential energies. While, in a recent paper (J. Chem. Theo.

Comput. 2023, 19 (11), 3063-3079), the diabatization could also achieved merely on the adiabatic energy data in constructing PES with neural networks.

4. The y axis in Figure 2 is claimed as logarithm of mean absolute errors in eV. It's quite confusing that the MAE could be such a large value between fitted PES and ab initio data.

Reviewer: 3

Comments to the Author

In this study, the authors propose a new method to learn the unsmooth adiabatic potential energy surfaces (PESs) associated with conical intersections (CIs). Given a simple mapping between the characteristic polynomial (CP) and the adiabatic potential energy, they find that it is possible to learn the smooth coefficients of and the average potential energy (ω-CP) instead of the unsmooth adiabatic potential energy. Furthermore, the authors find that the coefficient Cn-2(R) involves all the branching space information. By comparing the ω-CP method with the direct learning of the unsmooth adiabatic PES, the authors demonstrate the validity of their approach. They show that the advocated ω-CP method accurately describes the branch and seam space, the topography of CIs, and successfully reproduces all non-initial calculations in a variety of real and toy systems, outperforming the direct learning-based model. Overall, the authors present convincing evidence of the capability of their ω-CP method through tests on various systems. The manuscript is well written and the concept of learning the average potential energy is novel. I therefore recommend publishing this work. I have only a few minor comments below.

1. Considering the broad readership of JPCL, it would be better to give some specific explanations to some phrases, such as the seam space, branch space, etc.

2. The following sentence is not very clear, "Now, since the squared energy differences ΔE_ij (R) are lifted to second-order at a point of CI with respect to the branching space coordinates, and to fourth-order with respect to the remaining seam space coordinates, the Hessian". What are the meanings of the second order and fourth order here?

3. There is a typo in line 22 of page 11, where a redundant "vector" may be removed.

4. In SI, "These power spectra satisfy the above requirements of a descriptor, the proof and derivations can be found in (REF).". The "REF" should be referred to a specific reference.

Reviewer: 4

Comments to the Author

The manuscript presents an interesting approach to smooth representations of excited state energy landscapes in the branching space of a conical intersection. The authors show that this representation enables accurate and data-efficient representations of excited state energy landscapes up to and including the CI point. This is an interesting and highly useful approach. The manuscript provides

quantitative evidence that this outperforms direct learning. This manuscript in principle should be published, but the authors need to address some major and minor concerns described below.

- The statement "Given the central role played by CIs in photochemistry and photophysics, this poses a serious problem that previous works have not directly addressed" in the introduction should be reconsidered. Many of the cited previous works develop accurate and robust representations of excited states and couplings and show that the nonadiabatic dynamics are accurately reproduced. They do this despite describing the CI with lower accuracy, because the majority of nonadiabatic transitions occur in the vicinity of the CI and not directly at the CI. While this work shows that ab initio PES data can be accurately reproduced with this approach, it does not provide evidence that accurate dynamics simulations can be performed with it. The statement might be interpreted by the reader as suggesting that previously proposed methods are not able to faithfully reproduce landscapes and dynamics. I suggest expanding a bit more on what approaches have previously been proposed and what they have enabled rather than bulk referencing all recent literature at the end of this sentence.

- Connected to my previous point, the CP representation is ideal to represent the branching space and the coordinates close to the CI, but is it also applicable as a global representation of the PES? Judging from Figure 5, there is a point where the representation deviates from the ab initio data points. Far away from the CI, is the CP of Z numerically and analytically robust? If not, the approach would not be straightforwardly applicable for dynamics simulations (yet it would still be very useful for CI optimizations!) as it would not cover all the relevant phase space. This should be more clearly discussed.

- Is it really fair to only compare to direct learning of adiabatic PES? Nobody would do that anyway. As quasi-diabatic representations are effectively the state-of-the-art in ML for excited states, would it not be fairer to compare to that approach? I suggest that the authors also do that for at least one of their model systems. I am sure that their approach is superior, but by showing this explicitly, they show that their work does contribute something that goes beyond the current state of the art.

- First it is stated that the CP representation is not as useful as quasi-diabatic representations in the context of nonadiabatic dynamics as there is no direct access to nonadiabatic couplings. In the conclusions, it is then stated that there is no reason to use direct or quasi-diabatic approaches. This does not add up. The authors should provide a fair and clear explanation of the limitations of the approach and the scope of new capabilities it brings to the field.

- The manuscript is lacking computational details of the quantum chemistry calculations. Have they been previously published? If so it should be stated. Is the data publicly available? Are the ML models or the training data publicly available? If not, how would others be able to reproduce and verify the ML results? Please add the relevant data to ensure transparency. There are serious issues of reproducibility in the ML field and we should all strive to make it as easy as possible for others to build on our results.

- References are missing in the SI (placeholders)

Reviewer: 5

Comments to the Author

In this letter, Wang et al. introduced a ML method based on characteristic polynomial functions to construct multiple adiabatic PESs involving conical intersections. The potential matrix is split into an averaged energy matrix plus an energy-difference diagonal matrix. The latter is represented by its characteristic polynomials. Then, these coordinated-based CP coefficients and averaged potential energies are trained using a KRR model with a SOAP descriptor. Overall, I think this work provides some interesting findings; however, its few viewpoints cannot convince me with the present results. I suggest revision and addressing the following issues before considering its publication.

The potential matrix is split into an averaged energy matrix plus an energy-difference diagonal matrix, see Eq. 1. Such splitting indeed can lead to a smooth averaged energy matrix with respect to nuclear coordinates even at CIs. But, If I understood correctly, the possible non-smoothness at CIs should be transferred to the energy-difference diagonal matrix (if still in adiabatic representation), like a simple number decomposition of A=B+C. Please give some discussion on how this non-smoothness disappears in such simple splitting process.

Anyway, I understood this method is motivated by the early work by Domcke *et al.* in JPC, possible advantages and disadvantages should be discussed, as done by Domcke et al. in their

Summary and conclusions because of "no free lunch". They said in their JCP page 2, "It should be noted that matrix-diagonalization algorithms may be ill-conditioned in the case of nearly degenerate eigenvalues. The calculated eigenvalues therefore may not exactly reproduce the adiabatic PE surfaces in the immediate vicinity of conical intersections."

Therefore, although "The CP coefficients are also smooth functions of the nuclear coordinates over any domain, including one that contains a CI", there are possible non-smoothness problem at Cis when doing matrix-diagonalization. I would like to see some discussion on these possible points.

It is commonly known that the energy-based KRR method gives not-good gradients although good energies are gotten. In contrast, the gradient-KRR method provides good gradients, but not-good energies. This is very different from NN and DNN etc. which can provide both accurate energies and gradients, partly due to the use of both energies and gradients in the training process.

I suggest to add some tests on realistic systems in addition to test the first-order model of a two-state CI.

I did not agree with the point that the results from the KRR model can be simply transferred to neural networks, in particular more advanced ML models, e.g., DNN, etc. The KRR model is a simple combination of trained data with their coefficients to be trained while NN and DNN etc. are more suitable for super-linear problems. Recent work by Cui et al. has demonstrated that the DNN model can provide numerically accurate PES topologies around CIs if enough data are provided for the DNN training, see JPCL 2018, 9, 6702; 2018, 122,5660.

Overall, the present work provides new scheme to do PES fitting using ML techniques for multiple PESs involving CIs. But, there are some conclusion points to be modified or to be demonstrated with new data. Anyway, I would like to see its revised version.

Author's Response to Peer Review Comments:

Simon Neville
Michael Schuurman
National Research Council Canada
100 Sussex Drive
Ottawa, Ontario K1A 0R6
CANADA
Simon.Neville@nrc-cnrc.gc.ca
Michael.Schuurman@uottawa.ca

July 18, 2023

Re: Re-submission to The Journal of Physical Chemistry Letters

Dear Prof. Editor,

It is my pleasure to submit our revised version of the manuscript "Machine Learning Seams of Conical Intersections: A Characteristic Polynomial Approach". We thank the referees for their careful reading of the manuscript and for constructive comments on the work. In general, we have more strongly emphasized the novel elements of this work: an approach to learning smooth functions from which coupled adiabatic state manifolds can be extracted, without the need for an additional quasi-diabatization step. Furthermore, we also more clearly articulate our goal of quantitatively describing seams of conical intersection, in contrast to previous work which has successfully developed methods for fitting adiabatic surfaces and derivative couplings for trajectory simulations. These dynamics applications will be significantly less sensitive to the branching space topology, since the intersection is, in general, not accessed directly. In contrast, the optimization of conical intersections, for example, will be significantly more sensitive to seam of degeneracy the generation of CIs as opposed to an avoided crossings.

Each of referee comments and queries are addressed in order below.

Reviewer 1

1. *The method is interesting as sort of diabatization of adiabatic potential energy surfaces. The machine learning part is just routine work. Thus, I do not feel that the manuscript is suitable for publication on The Journal of Physical Chemistry Letters.*

   The authors thank the referee for their comments. To clarify, we believe one of the main strengths of the current work is that it provides a route to learn smooth functions *without* requiring a diabatization step that will in general be approximate and not uniquely defined. Lastly, the authors agree that the technical elements of the learning procedure are not novel, but consider this a feature of the current proposal and not a shortcoming. Specifically, standard kernels and classical ML approaches can be successfully employed given the choice of target values (i.e. characteristic polynomial coefficients).

Reviewer 2

1. *The model system in current work only covers CI region. However, as the future of CP model is build PES for nonadiabatic molecular dynamics simulation, the moderate example would*

naturally covers an entire relaxation pathway, i.e. double bond twisting of ethylene. The difficulty for fitting PESs coupled with CIs not only the CI region itself but also the switching area toward CI. Moreover, the authors claimed that the CP based PESs could extrapolated to area faraway from training set geometries, it is quite interesting that whether the CP model could build PES with sparser ab initio geometry grids?

Indeed, to be more broadly useful for dynamics and structure optimization, surfaces that describe both the approach and exit from the CI coupling region will be required. This will be the focus of future work. In this initial study, we have chosen to focus on a particularly difficult aspect of this problem: the description of the seam space in which the potential surfaces are not differentiable to any order. We conceive of the present work as a confirmation that the $\omega-$CP approach is worth pursuing and intend to explore extended regions of the potential surfaces in an upcoming work.

That said, and as the referee notes, the CP coefficients are slowly varying over large regions of coordinate space, which makes them amenable to extrapolation. This is shown implicitly in Figure 2, where it is observed that relatively sparse grids can yield surfaces of chemical accuracy. To more explicitly convey this, we have now included in the SI (Table S7) the dependence of the branching space topology (the focus of this work) on the number of fit points for the accidental conical intersection in ethylene. We have added the following text to the main body of the manuscript:
In fact, the ω-CP models are able to reproduce the branching space with very modest training set sizes, as shown in Table S7 in the SI, achieving reasonable accuracy with set sizes as small as 500 *ab initio* points.

2. *In current paper, only the fitted adiabatic potentials were presented, the performance of energy gradients with CP based PESs are not tested, which is of crucial importance to ensure trajectory propagation on PESs follow reasonable reaction pathways.*

The present work is focused on the description of the branching space of a conical intersection, which will be important for a quantitative description of nonadiabatic dynamics, but wave packet dynamics is not the focus of the present work. The ability of these potentials to reproduce the energy gradients of the adiabatic potentials will be the basis of a future work.

That said, the current results do imply high accuracy in the description the gradients of the adiabatic potentials. Specifically, from the calculation of the eigenvalues of the c^Z_{n-2} Hessian in Eq. (5). The quantitative reproduction of these quantities requires an accurate description of the gradients of the adiabatic potentials as c^Z_{n-2} corresponds to the *squares* of the adiabatic energy differences. Thus the second-derivatives of this quantity encodes information on the gradients of the potential. While this analysis is only performed at the point of conical intersection, the CI corresponds to the point on the surface where a reproduction of the gradients would be considered most difficult.

3. *In Page 14, the authors have addressed one of the advantage of CP model compared with diabatic PES is free of additional calculations beyond potential energies. While, in a recent paper (J. Chem. Theo. Comput. 2023, 19 (11), 3063-3079), the diabatization could also achieved merely on the adiabatic energy data in constructing PES with neural networks.*

The authors thank the referee for drawing our attention to this recent publication, we now cite this work and others concerning diabatization by neural networks. However, the intent of the present work is not to claim that this approach is unique in the ability to learn seams

of conical intersection using adiabatic energies alone, but rather, we demonstrate that this is a comparatively simple and efficient (see Figure 2) approach to do so. Moreover, we note that a diabatization by *ansatz* using a neural network, as discussed in the above reference, inherently requires the imposition of constraints. Namely, the enforcement of zero diabatic couplings at some arbitrary point in nuclear configuration space in order to fix the global gauge of the adiabatic-to-diabatic transformation. This requires additional user insight and input beyond the calculation of adiabatic potential energies. In contrast, the ω-CP approach is uniquely defined and requires no user input in the form of constraints.

We have added the following text to the introductory section of the manuscript:

One approach to circumvent this issue is to instead learn diabatic potentials, which are expected to be smooth functions of nuclear coordinates. This procedure will in general, however, require a (non-unique) choice as to how the surfaces will be diabatized, and typically requires user-input and intuition beyond the computation of electronic energies.

4. *The y axis in Figure 2 is claimed as logarithm of mean absolute errors in eV. It's quite confusing that the MAE could be such a large value between fitted PES and ab initio data.*

   Figure 2 displays the Log(MAE) for the direct fitting of adiabatic energies and the ω-CP method. Note that the y-axis values are negative, so that a y-value of "-3" corresponds to an MAE of 0.001 eV. Indeed, so-called "chemical accuracy" of 1 kcal mol^{-1} is indicated by a dashed line in each of the panels. This is approaching spectroscopic accuracy (1 cm^{-1}), which greatly exceeds the requisite accuracy for excited-state dynamics and many spectroscopic applications.

Reviewer 3

1. *Considering the broad readership of JPCL, it would be better to give some specific explanations to some phrases, such as the seam space, branch space, etc.*

   The authors thank the referee for this suggestion. We have added definitions of these terms, as well as a description of their physical significance to the Introduction on pg. 2. We have added the following text:
   ...of the branching space. Conical intersections are not isolated points, but rather, form connected seams of degeneracy. At a point of two-state conical intersection, this degeneracy is lifted at first-order in precisely two directions, and this two-dimensional subspace is termed the *branching space*. Necessarily, then, the degeneracy will be preserved at first-order in the remaining $3N-8$ internal coordinates, collectively denoted the *seam space*.

2. *The following sentence is not very clear, "Now, since the squared energy differences $\Delta E_{ij}(R)$ are lifted to second-order at a point of CI with respect to the branching space coordinates, and to fourth-order with respect to the remaining seam space coordinates, the Hessian". What are the meanings of the second order and fourth order here?*

   The electronic energy in the vicinity of a conical intersection is typically described using a perturbation theory analysis. The "orders" referenced here quantify the extent to which a particular quantity (eg. the energy difference) depends on changes to a particular variable (in this case, displacements from the CI). So, for example, the degeneracy at a CI is lifted at "first-order" with respect to displacements from the CI in the branching space: the potential curves are linear and with non-zero slope as one moves away from the CI along the branching space

coordinates. Therefore, referring to the quantities above, the *difference* in energy between the two states (which is zero at the point of CI) increases quadratically (eg. at "second-order") with respect to displacements from the CI in the branching space. Since the seam space coordinates *preserve* the degeneracy at first order, these same quantities will only affect this quantities at higher orders (eg. larger displacements).

3. *There is a typo in line 22 of page 11, where a redundant "vector" may be removed.*

   The authors thank the referee for catching this error. It has been corrected.

4. *In SI, "These power spectra satisfy the above requirements of a descriptor, the proof and derivations can be found in (REF).". The "REF" should be referred to a specific reference.*

   The missing reference has been replaced with the following:
   Bartok, A. P., Kondor, R., Csanyi, G. On representing chemical environments. *Phys. Rev. B*, **87**, 184115, (2013).

Reviewer 4

1. *The statement "Given the central role played by CIs in photochemistry and photophysics, this poses a serious problem that previous works have not directly addressed" in the introduction should be reconsidered. Many of the cited previous works develop accurate and robust representations of excited states and couplings and show that the nonadiabatic dynamics are accurately reproduced. They do this despite describing the CI with lower accuracy, because the majority of nonadiabatic transitions occur in the vicinity of the CI and not directly at the CI. While this work shows that ab initio PES data can be accurately reproduced with this approach, it does not provide evidence that accurate dynamics simulations can be performed with it. The statement might be interpreted by the reader as suggesting that previously proposed methods are not able to faithfully reproduce landscapes and dynamics. I suggest expanding a bit more on what approaches have previously been proposed and what they have enabled rather than bulk referencing all recent literature at the end of this sentence.*

   The authors thank the referee for their comment, and are in agreement with their assessment: trajectory-based nonadiabatic dynamics methods, surface hopping in particular, are likely to be much less sensitive to the branching space topology since they in general do not access the CIs directly, but rather, only their vicinity. If the nonadiabatic coupling is particularly large, as is the case in ultrafast electronic relaxation, the surface "hops" may occur at significant distances from CI and involve relatively large energy gaps between the relevant electronic states.

   However, the comment the referee references was intended to refer specifically to learning of conical intersections and the corresponding branching and seam spaces. Certain applications, such as the optimization of conical intersection structures, will necessarily be more sensitive to the presence (or lack thereof) of a true point of degeneracy.

   To better clarify our intent, we have amended the above passage to read as the following:
   Given the central role played by CIs in photochemistry and photophysics, the ability of ML surrogate potentials to quantitatively reproduce the branching and seam-space topographies is a problem previous works have not directly addressed.

2. *Connected to my previous point, the CP representation is ideal to represent the branching space and the coordinates close to the CI, but is it also applicable as a global representation of the PES? Judging from Figure 5, there is a point where the representation deviates from the ab initio data points. Far away from the CI, is the CP of Z numerically and analytically robust? If not, the approach would not be straightforwardly applicable for dynamics simulations (yet it would still be very useful for CI optimizations!) as it would not cover all the relevant phase space. This should be more clearly discussed.*

   The authors are confident that this approach will be applicable to all regions of the PES, not just the CI region. This work focuses on the a region of CI since it will be the most challenging region of the manifold to learn given the non-differentiability of the potentials. In regions far removed from the CI, the adiabatic surfaces will also be smooth and the CP-coefficients, being simple sums of products of the former, will be smooth as well. The purpose of this letter is to highlight the success of the approach on what the authors feel is a particularly challenging (but relevant) problem that to date has not received significant attention: the reproduction of the branching and seam spaces of a conical intersection. Given the success of the approach on this difficult problem, the authors are encouraged to proceed in the development of the approach for more general problems. In fact, Ref. 20 shows quantitative agreement over large regions of coordinate space for a molecular example.

   Regarding the numerical stability of the CP-coefficients: the working equations are numerically robust and do not contain any singularities. Therefore, there is no reason to think that the agreement we see here is dependent on the proximity to the CI. However, if the referee is referring to the deviation between the CP-coefficient derived surrogate potentials and the *ab initio* surfaces shown in Figure 5, the authors wish to emphasize that this is due the extrapolation of the CP-coefficient potentials to regions far removed from the nuclear structures in the training set, and should not be interpreted as a failure of the CP-coefficients to reproduce training data.
   We have amended Figure 5 to show the extent of the fit data in an effort to anticipate a potential misinterpretation of the Figure. The figure caption now reads:

   Adiabatic potentials over an extended region of coordinate space along the (rectilinear) branching space directions, x and y, for the two-state examples. The shaded regions show the energetic extent of the fit set used to generate the direct and ω-CP models. The ω-CP models exhibit impressive fidelity to the *ab initio* surfaces over an energy range significantly exceeding that of the fit data.

3. *Is it really fair to only compare to direct learning of adiabatic PES? Nobody would do that anyway. As quasi-diabatic representations are effectively the state-of-the-art in ML for excited states, would it not be fairer to compare to that approach? I suggest that the authors also do that for at least one of their model systems. I am sure that their approach is superior, but by showing this explicitly, they show that their work does contribute something that goes beyond the current state of the art.*

   There are many examples of the direct learning of adiabatic potentials in the recent literature (see Refs. *J.Phys.Chem.Lett.*,**2018**,9, 2725–2732, *J. Chem. Phys.*, **2018**, 147, 084105, *Mach. Lean.: Sci. Technol.*, **2021**, 2, 035039). In fact, the authors agree that learning adiabatic potentials should be avoided, and is one of the main points of the current work. The current approach allows for quantitative descriptions of CI regions, where the direct fitting of adiabatic

energies fails, but requiring as input only the adiabatic potentials themselves (i.e. the same "ingredients").

However, the authors agree that the ability to efficiently learn diabatic potentials has many advantages, furnishing, for example, nonadiabatic couplings in addition to the potential surfaces. Indeed, a detailed comparison to current approaches to fitting these quantities would be interesting. However, firstly, the purpose of this work is to address the practice of directly learning adiabatic potential energy surfaces to describe intersecting potentials; an approach that is significantly improved via the current work. Secondly, learning a 'quasi-diabatic' representation is complicated by the fact this object is not uniquely defined, and thus any comparison would require the selection of a particular diabatization approach. That said, the authors are in agreement that this comparison would be informative and worth pursuing in a future work. To more explicitly state the goals of the current work we have changed the following wording:
In this Letter, we demonstrate that accurate ML models of CI seams, including a correct description of the branching space, can in fact be constructed using just adiabatic electronic energies by forgoing the direct learning of PESs. Instead, we advocate for the learning of the nuclear coordinate-dependent coefficients of the characteristic polynomial (CP) of a simple decomposition of the potential matrix.

4. *First it is stated that the CP representation is not as useful as quasi-diabatic representations in the context of nonadiabatic dynamics as there is no direct access to nonadiabatic couplings. In the conclusions, it is then stated that there is no reason to use direct or quasi-diabatic approaches. This does not add up. The authors should provide a fair and clear explanation of the limitations of the approach and the scope of new capabilities it brings to the field.*

   In the conclusion the authors state: "Here, there seems to be no reason to continue using directly-learned adiabatic PESs, given the unambiguous advantages of the indirect ω-CP approach.". Here, we are referring to the direct learning of *adiabatic* surfaces, not diabatic potential matrices. As stated above, the main advance of the present work is a method to accurately learn seams of conical intersection using only adiabatic energies.

5. *The manuscript is lacking computational details of the quantum chemistry calculations. Have they been previously published? If so it should be stated. Is the data publicly available? Are the ML models or the training data publicly available? If not, how would others be able to reproduce and verify the ML results? Please add the relevant data to ensure transparency. There are serious issues of reproducibility in the ML field and we should all strive to make it as easy as possible for others to build on our results.*

   The details of the calculations are described in the Supporting Information. An additional section has been added to the SI under **ML computational details** that contains all relevant quantities to reproduce the ML models including KRR kernel and SOAP (hyper)parameters. However, to give a sense of the level of theory employed, we have added the following text to the manuscript on pg. 6:
   The adiabatic energies that comprised the training sets were computed at the multireference configuration interaction (MRCI) level of theory, in which the reference was generated from a minimum orbital complete active space (CASSCF) wave function optimization using the COLUMBUS electronic structure package. The nuclear structures were generated via Latin hypercube sampling about each MECI geometry. Further details are provided as supporting information.

6. *References are missing in the SI (placeholders)*

   The authors thank the referee for this comment and have added the appropriate reference: Bartok, A. P., Kondor, R., Csanyi, G. On representing chemical environments. *Phys. Rev. B*, **87**, 184115, (2013).

Reviewer 5

1. *The potential matrix is split into an averaged energy matrix plus an energy-difference diagonal matrix, see Eq. 1. Such splitting indeed can lead to a smooth averaged energy matrix with respect to nuclear coordinates even at CIs. But, If I understood correctly, the possible non-smoothness at CIs should be transferred to the energy-difference diagonal matrix (if still in adiabatic representation), like a simple number decomposition of A=B+C. Please give some discussion on how this non-smoothness disappears in such simple splitting process. Therefore, although "The CP coefficients are also smooth functions of the nuclear coordinates over any domain, including one that contains a CI", there are possible non-smoothness problem at CIs when doing matrix-diagonalization. I would like to see some discussion on these possible points*

   As stated by the referee, a simple decomposition of the potential matrix doesn't guarantee the splitting matrix Z to be smooth with respect to nuclear coordinates. This is where the Characteristic Polynomial (CP) of the splitting matrix is used: The CP coefficients of the splitting matrix *are* smooth, rendering it amenable for learning. The splitting matrix elements can then be trivially obtained from the CP coefficients by diagonalizing the companion matrix in equation 5.

   That the coordinate dependence of these coefficients will in general be smooth is difficult to prove definitively, however, we emphasize that the CP coefficients of the potential matrix are invariant with respect to unitary transformation of this quantity. Therefore, CP-coefficients determined from the adiabatic potential matrix will be *identical* to those for a potential matrix that has been rotated to yield, for example, a set of diabatic electronic states. Therefore, the fact that CP-coefficients are smooth functions of nuclear coordinates is not a result of the representation of the potential matrix, but rather, is an inherent property of the characteristic polynomial. To emphasize this point, we have added the following text to pg. 4:
   ...that contains a CI. Given that the characteristic polynomial of a given matrix is invariant to similarity transformations, the CP coefficients of the splitting matrix Z are invariant with respect to the choice of electronic basis. Thus, for example, the same set of CP coefficients are generated in the adiabatic representation as for a diabatic representation. The same holds for the $\omega(R)$ term, being as it is the trace of the potential matrix.

2. *It is commonly known that the energy-based KRR method gives not-good gradients although good energies are gotten. In contrast, the gradient-KRR method provides good gradients, but not-good energies. This is very different from NN and DNN etc. which can provide both accurate energies and gradients, partly due to the use of both energies and gradients in the training process. I suggest to add some tests on realistic systems in addition to test the first-order model of a two-state CI.*

   The current results do imply high accuracy in the description the gradients of the adiabatic potentials. Specifically, from the calculation of the eigenvalues of the c^Z_{n-2} Hessian in Eq. (5). The quantitative reproduction of these quantities requires an accurate description of the

gradients of the adiabatic potentials as c_{n-2}^{Z} corresponds to the *squares* of the adiabatic energy differences. Thus the second-derivatives of this quantity encodes information on the gradients of the potential. While this analysis is only performed at the point of conical intersection, the CI corresponds to the point on the surface where a reproduction of the gradients would be considered most difficult.

Furthermore, to clarify, the data in this manuscript was generated using *ab initio* electronic structure computations; in this report the ω-CP approach is employed to learn *ab initio* potential surfaces, not first-order models. The fact the degeneracy is lifted at first-order is an inherent property of a conical intersection, and should not be interpreted as the application of an empirical/toy model.

3. *I did not agree with the point that the results from the KRR model can be simply transferred to neural networks, in particular more advanced ML models, e.g., DNN, etc. The KRR model is a simple combination of trained data with their coefficients to be trained while NN and DNN etc. are more suitable for super-linear problems. Recent work by Cui et al. has demonstrated that the DNN model can provide numerically accurate PES topologies around CIs if enough data are provided for the DNN training, see JPCL 2018, 9, 6702; 2018, 122,5660.*

   We thank the referee for pointing out these references, we now include it in this manuscript. The phrase identified by the referee previously stated: "although the conclusions drawn should be transferable to other ML methods, e.g., the construction of neural network potentials.". The authors agree that this statement is ambiguous, and could be interpreted as meaning the direct learning of adiabatic energies, using any approach and training set size, will in general not be able to reproduce the branching space of a conical intersection. This was not our intent. We mean simply that the *methodology* adopted here can be applied to more advanced ML techniques. Specifically, the smooth-ness of the CP-coefficients will be amenable to learning using a variety of ML approaches. We have changed the sentence in question to read as follows:

   although the methodology adopted here will be transferable to other ML methods.

   as well as the following to the concluding statement:

   This may be overcome by switching to a diabatization by ansatz, neural network-based implementations of which have recently been reported. However, such an approach requires additional, non-uniquely defined user-defined constraints to be imposed on the model quasi-diabatic potentials.

jz-2023-01649z.R2

Name: Peer Review Information for "Machine Learning Seams of Conical Intersection: A Characteristic Polynomial Approach"

Second Round of Reviewer Comments

Reviewer: 4

Comments to the Author

I am happy with the revisions the authors have made and recommend publication.

Reviewer: 1

Comments to the Author

I do not think that revised manuscript properly answer questions addressed by referees.

Reviewer: 2

Comments to the Author

The ingenious algorithm proposed in current paper could convert the sloped PES toward conical intersection into moderate from which is benefit for machine learning. Although the examples only applied for simple two-state conical region, this method do worth trying for researcher to construct accurate multi-state PES by a convenient way.

The authors have addressed convincing improvements in current version. I suggest this paper to be published with a minor revision. The ab initio methods in SI need to be clarified, ie.by which method the CI structures were optimized? State-averaged CASSCF or MRCI? If optimized by CASSCF and correcting energy by MRCI, the degenerated energy at CI point in Fig.4 is confusing.

Author's Response to Peer Review Comments:

Simon Neville
Michael Schuurman
National Research Council Canada
100 Sussex Drive
Ottawa, Ontario K1A 0R6
CANADA
Simon.Neville@nrc-cnrc.gc.ca
Michael.Schuurman@uottawa.ca

August 9, 2023

Re: Re-submission to The Journal of Physical Chemistry Letters

Dear Prof. Editor

It is our pleasure to submit our revised version of the manuscript "Machine Learning Seams of Conical Intersections: A Characteristic Polynomial Approach". We have addressed the remaining comments from the reviewers as well as those from the editorial office, as shown in detail below.

Sincerely,
Simon Neville
Michael Schuurman

Reviewer 2

1. *The ab initio methods in SI need to be clarified, ie.by which method the CI structures were optimized? State-averaged CASSCF or MRCI? If optimized by CASSCF and correcting energy by MRCI, the degenerated energy at CI point in Fig.4 is confusing.*

   The section "Quantum Chemistry Calculations" in the SI present further details of the electronic structure computations, including the state-averaging scheme for each of the molecules in the study. Note that all MECI optimizations and energy computations were performed at the same (MRCI) level of theory.

Editorial Office

1. *Please resize the TOC graphic per journal guidelines (2in x 2in).*

   This has been done.

2. *In both the main file and the supporting information, fix the style of all references to use JPCL formatting (check all references carefully).*

   All references are now in JPCL format.

3. *Please add the postal code to the first affiliation on the title page of the SI file.*

   This has been done.

4. *"Figures S4 and S5" are cited in the manuscript text, but there is no Figure S5 present in the SI file. Please add the missing Figure S5 to the SI file or correct the citation in the manuscript.*

   The reference to Figure S5 has been removed from the text

5. *lease include annotated version(s) of your revised publication file(s) with colored text or highlights indicating the revisions that you have made,*

   This file is now included as main_annotated.pdf.
